# Correction: Dutta et al. Transcriptional Regulation of CCL2 by PARP1 Is a Driver for Invasiveness in Breast Cancer. *Cancers* 2020, *12*, 1317

**DOI:** 10.3390/cancers16010007

**Published:** 2023-12-19

**Authors:** Pranabananda Dutta, Kimberly Paico, Gabriela Gomez, Yanyuan Wu, Jaydutt V. Vadgama

**Affiliations:** 1 Division of Cancer Research and Training, Department of Medicine, Charles R. Drew University of Medicine and Science, Los Angeles, CA 90059, USA; pranabandutta@cdrewu.edu (P.D.); kpaico@ucmerced.edu (K.P.); gabrielagomez@cdrewu.edu (G.G.); yanyuanwu@cdrewu.edu (Y.W.); 2 Jonsson Comprehensive Cancer Center, David Geffen School of Medicine, The University of California at Los Angeles, Los Angeles, CA 90059, USA

## Error in Figure

In the original publication, there was a mistake in Figure 5D as published [1]. The top left and the bottom left panels were inadvertently duplicated. The corrected Figure 5D panel appear below.

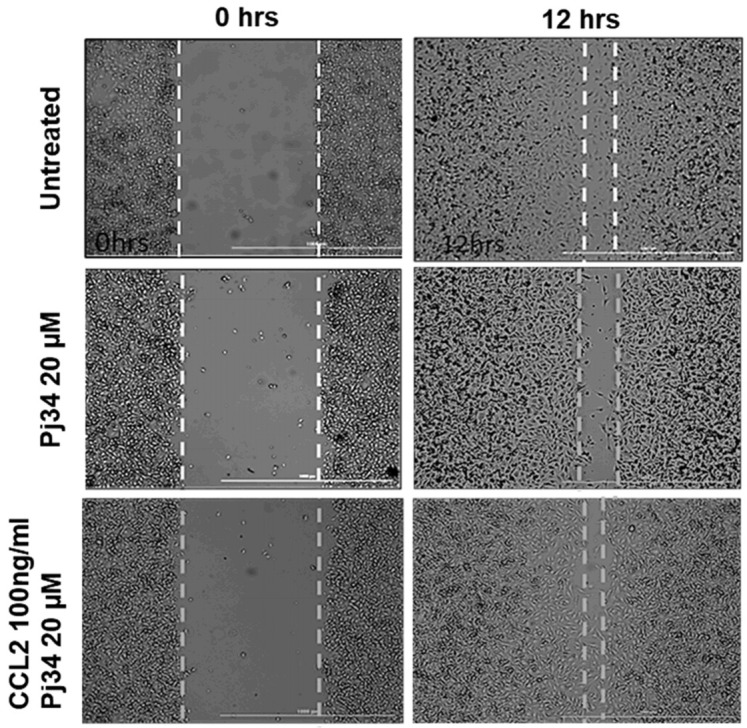



The full Figure 5 appear following.

The authors apologize for any inconvenience caused and state that the scientific conclusions are unaffected. This correction was approved by the Academic Editor. The original publication has also been updated.

## Figures and Tables

**Figure 5:**
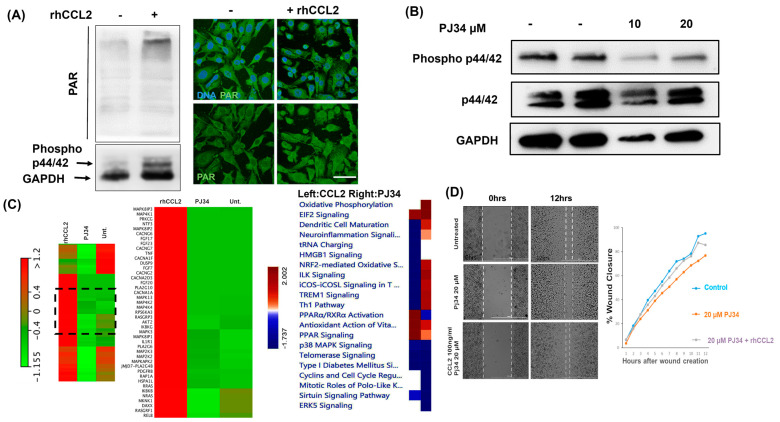
**PARP1 and CCL2 crosstalk regulate invasiveness in breast cancer.** (**A**) Left: Recombinant human CCL2 (rhCCL2) can increase PAR levels in MB-231 cells. MB-231 cells were serum-starved overnight and treated with 50 ng/mL rhCCL2 for 1 h. Western blot showing total PAR level upon treatment. Right: Immunofluorescence images showing PAR (green) in cells after rhCCL2 treatment. An enlarged image in Figure S8. Merged image with DNA (DAPI) shown on top. Scale bar 20 µm. (**B**) PARP1 inhibitor treatment reduces phosphorylated P44/42 (ERK1/2) levels in MB-231cells. Western blot showing Phospho-p44/42 ERK1/2 (Thr202/Tyr204) and total ERK1/2 after overnight PJ34 treatment followed by 45 min of TNFα. (**C**) Left: Heat map showing transcriptome analysis from MB-231 cells for rhCCL2 (1 h treatment as in (**A**)) and PJ34 (10 µM Overnight treatment) treated groups. The KEGG MAP kinase pathway genes are identified. Red color indicates upregulation with green, showing the downregulation of mRNA transcription. Right: Ingenuity pathway analysis from rhCCL2 (Left column) and PJ34 treated (right column). Enlarged images of (**C**) are in Figure S8. (**D**) Wound healing assay with simultaneous rhCCL2 and PJ34 treatment on MB-231 cells. Near confluent cells were pretreated with PJ34 before rhCCL2 was added at the dose of 50 ng/mL after wound creation. Treatment was continued for 12 h. The graph on the right shows % wound closure with respect to time. Scale bar 100 µm. Whole western blots for (**A**,**B**) are in Figure S10.
