# Supplementary material for: Transcriptional Regulation of CCL2 by PARP1 Is a Driver for Invasiveness in Breast Cancer
Source: Cancers (Basel). 2020 May 21;12(5):1317. doi: 10.3390/cancers12051317 (PMC7281677; doi:10.3390/cancers12051317)
Supplement: Supplementary file 1 [file cancers-12-01317-s001.zip › cancers-789140-Supplementary materials-figures.pdf]

Supplementary Materials: Transcriptional Regulation of *CCL2* by PARP1 Is a Driver for Invasiveness in Breast Cancer

Pranabananda Dutta, Kimberly Paico, Gabriela Gomez, Yanyuan Wu and Jaydutt V. Vadgama

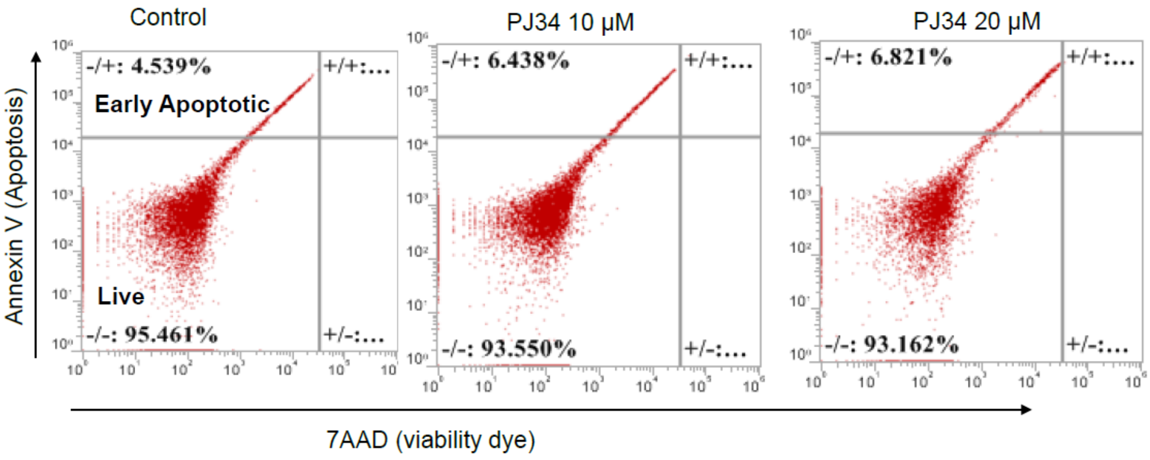

Figure S1. Overnight treatment with PJ34 does not cause significant apoptosis. Annexin V positive cells are considered early apoptotic (Top left quadrant). Annexin V and 7AAD positive cells are late apoptotic (Top right quadrant). Bottom left quadrant: 7AAD negative are live cells. Bottom right quadrant: 7AAD positive cells are possibly necrotic/dead.

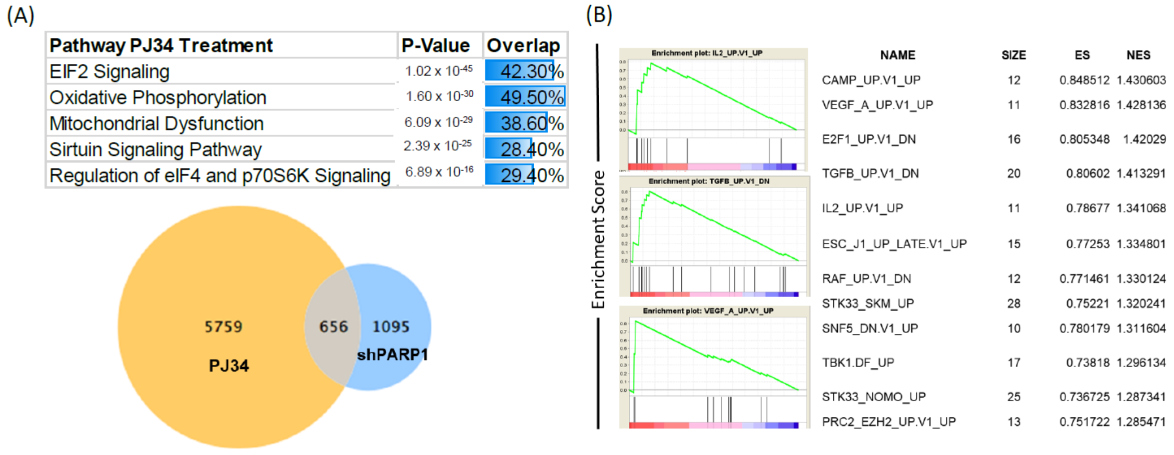

Figure S2. (**A**) Top: Pathways affected by PJ34 treatment in MB-231 cells. Bottom: Venn diagram of differentially expressed genes in MB-231 cells after PARP1 inhibition with PJ34 and shRNA mediated knockdown in BT549 cells. (**B**) Gene Set Enrichment Analysis (GSEA) on the 656 overlapping genes from (**A**) between PARP1 inhibition and knockdown is shown. Analysis was performed on the MsigDB oncogenic signature (c6.all.v6.2.symbols.gmt). Name: Gene expression set name from MsigDB. Size: number of genes belonging to the particular gene set from the list of 656 genes. ES = Enrichment Scores (in green line) (Number showing the degree to which the gene is overrepresented in the dataset)., NES = Normalized Enrichment Score [Actual ES/mean (ES against all permutation of the dataset)], middle lines shows member of the gene sets found in the gene list provided. The red to blue bar along *x*-axis denotes positive (red) to negative (blue) enrichment. The lines also represent leading edge subsets. From: "https://www.gsea-msigdb.org/"

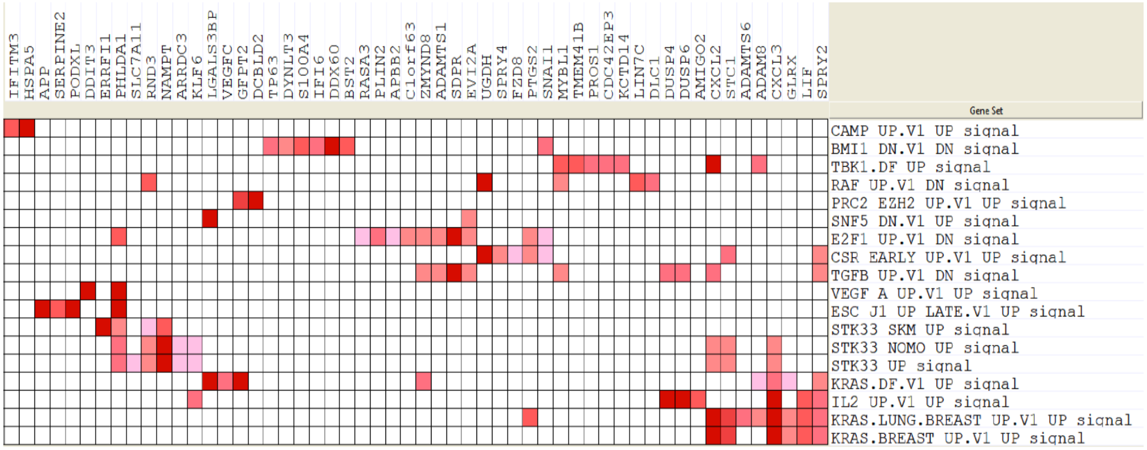

Figure S3. GSEA leading-edge analysis on significant Gene sets from PJ34-PARPi with 656 overlapping genes. The leading edge subset of genes is shown after enrichment as found in the GSEA analysis using pre-ranked method. Clustering of genes was removed for clarity. Heatmap is showing log2 fold change expression rank values of the genes listed on the column. Genes set are shown on the rows of the heatmap. The colors on the heatmap shows range of expression values with darker color representing higher log2 fold expression difference.

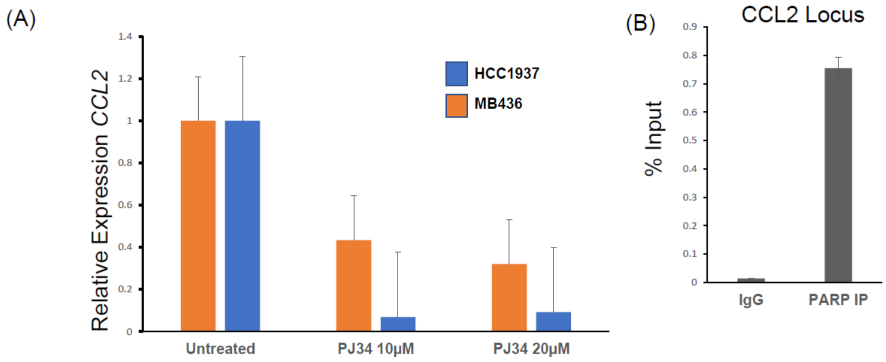

Figure S4. Downregulation of *CCL2* upon PARP1 inhibitor Treatment in BRCA negative TNBC cells. (**A**) Overnight Treatment with PJ34 in BRCA1 negative TNBC breast cancer cells. (**B**) Chromatin IP with PARP1 antibody in BRCA1 negative HCC1937 at the *CCL2* promoter under basal condition without any treatment.

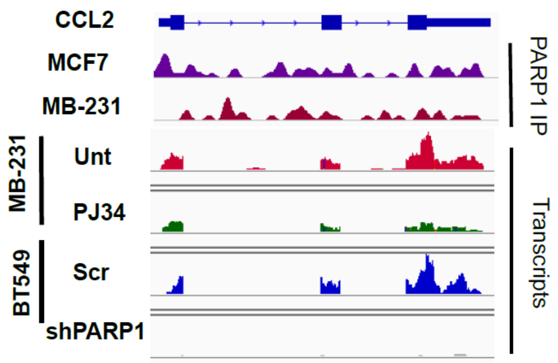

Figure S5. PARP1 localization across the CCL2gene locus on chromosome 17. IGV genome browser (http://software.broadinstitute.org/software/igv/) screenshot showing PARP1 recruitment in MCF7 and MB-231 cells at the *CCL2* locus. Data from GSE61916 shows PARP1 recruitment at the *CCL2* gene on Chromosome 17. Bottom rows showing RNA transcriptome coverage with PJ34 treatment (MB-231) or PARP1 knockdown (BT549).

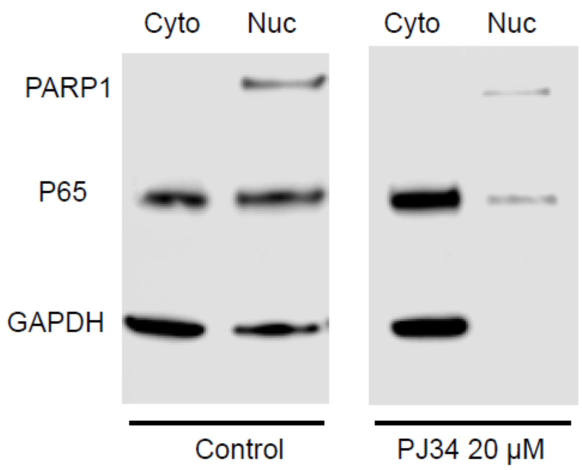

Figure S6. PARP1 inhibition reduces nuclear P65. Cytoplasmic (Cyto), nuclear (Nuc), distribution of PARP1 and P65 with or without overnight PJ34 treatment.

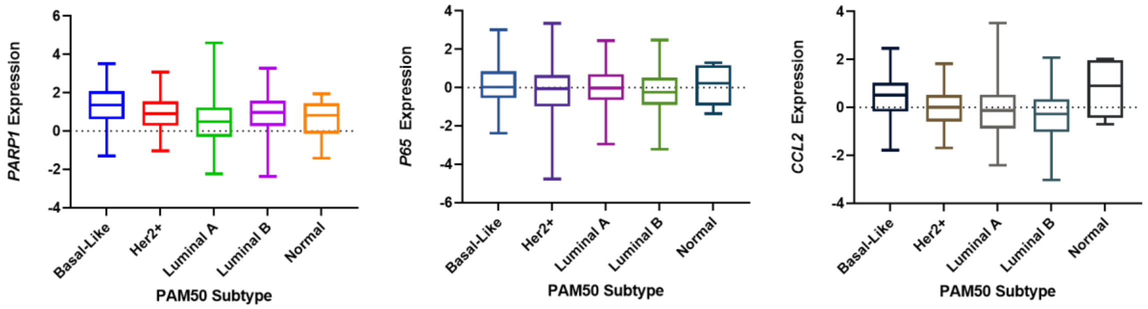

PARP1 Expression

Basal-Like vs. Luminal A	*$p < 0.001$
Basal-Like vs. Luminal B	*$p < 0.0123$

CCL2 Expression

Basal-Like vs. Luminal A	*$p < 0.0001$
Basal-Like vs. Luminal B	*$p < 0.0001$

Figure S7. Expression of PARP1, P65, CCL2 from TCGA breast cancer dataset. * Tukey's multiple comparison, One way ANOVA Data from UC Santa Cruz xenabrowser.net TCGA Breast Cancer (BRCA).

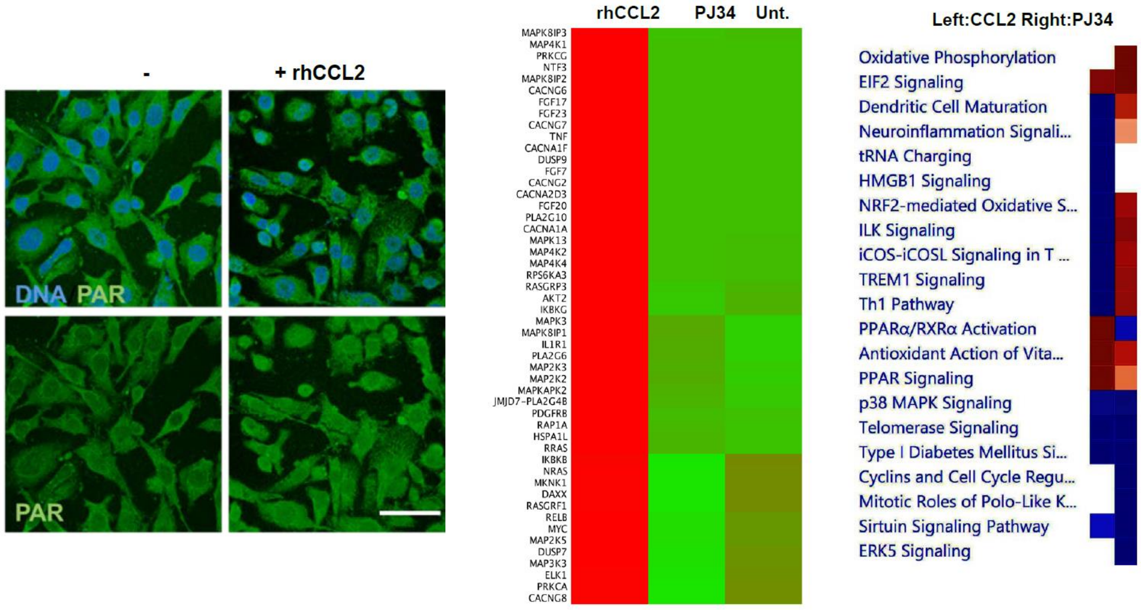

Figure S8. Enlarged images of Figure 5A and 5C. Scale bar left panel: 20 µm.

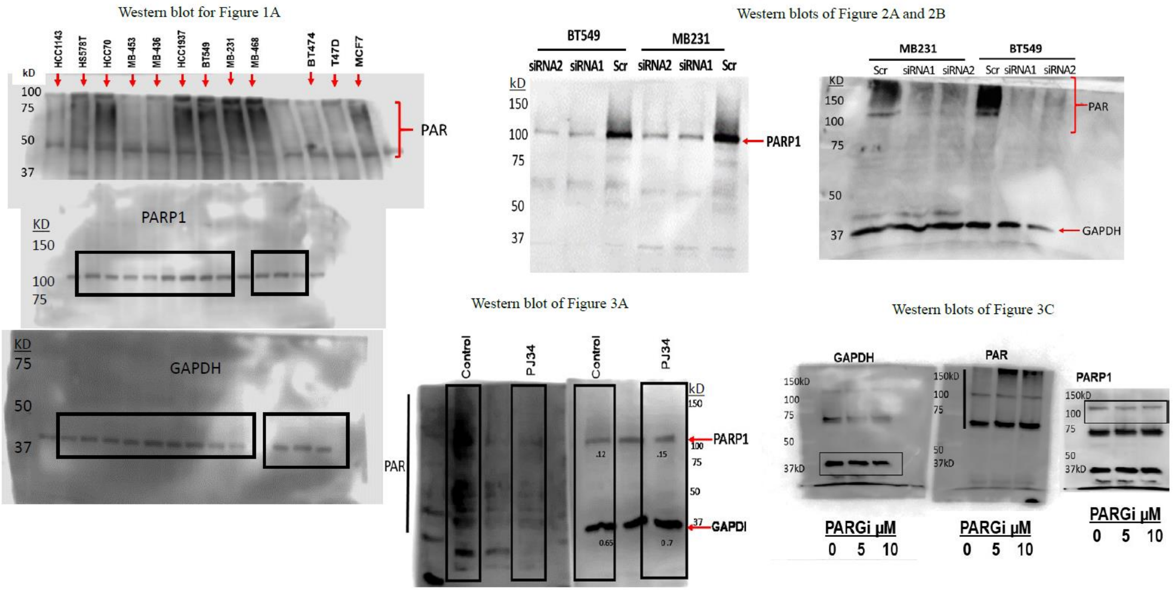

Figure S9. Whole western blots in Figure 1 to Figure 3.

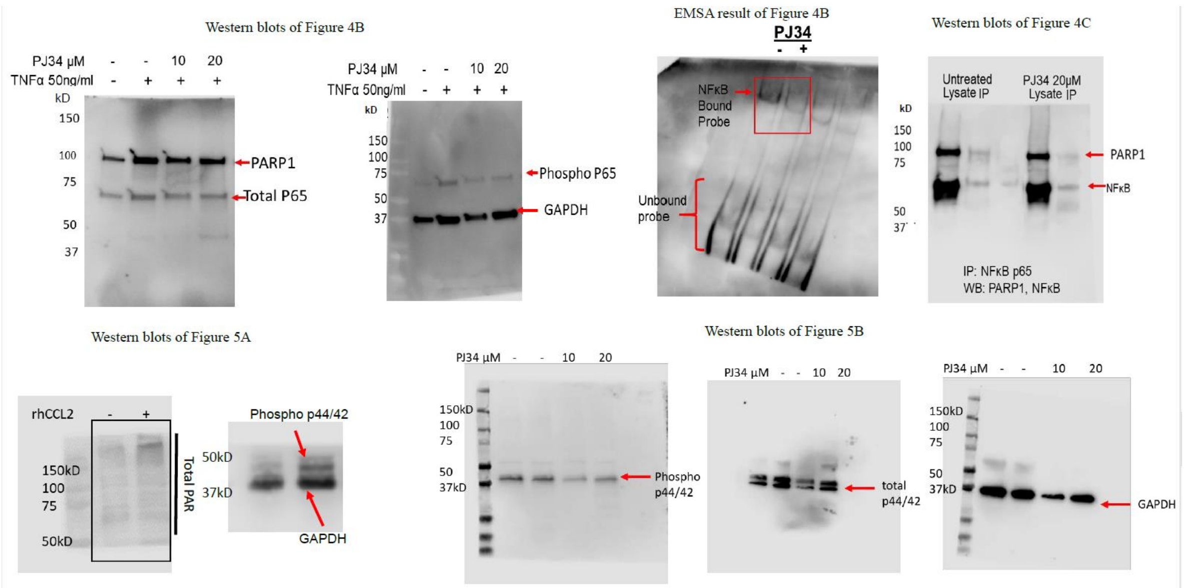

Figure S10. Whole western blots in Figures 4 and 5.

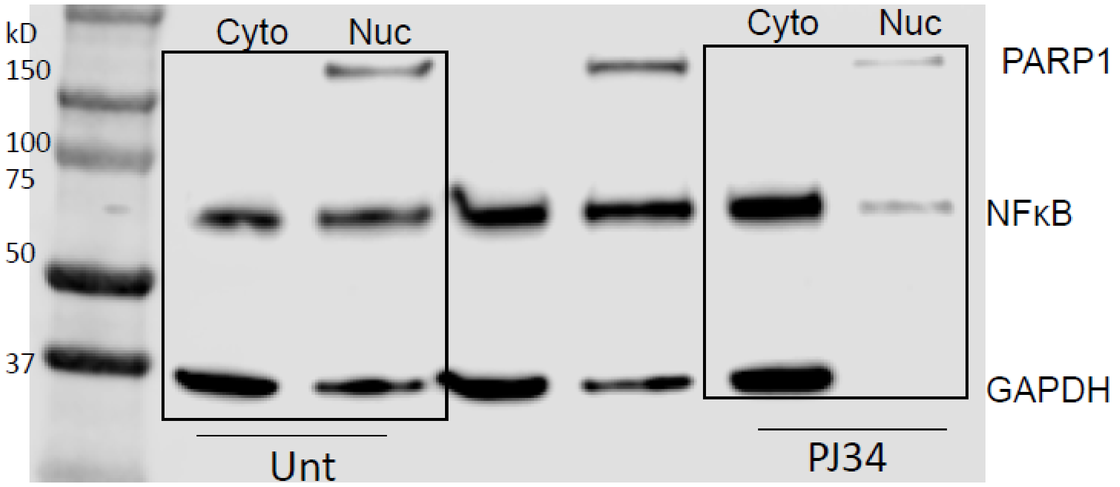

Figure S11.Whole western blots in Figure S6

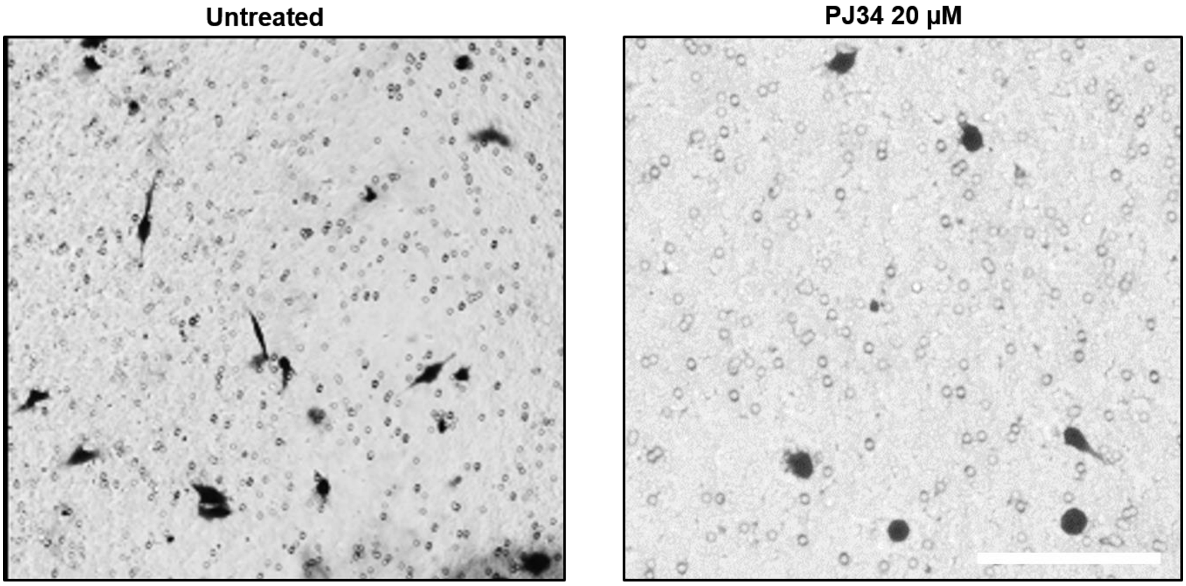

Figure S12. Enlarged view of Figure 1C Boyden chamber assay. Scale Bar 10 μm.
